# A High Affinity Red Fluorescence and Colorimetric Probe for Amyloid β Aggregates

**DOI:** 10.1038/srep23668

**Published:** 2016-04-01

**Authors:** K. Rajasekhar, Nagarjun Narayanaswamy, N. Arul Murugan, Guanglin Kuang, Hans Ågren, T. Govindaraju

**Affiliations:** 1 Bioorganic Chemistry Laboratory, New Chemistry Unit, Jawaharlal Nehru Centre for Advanced Scientific Research, Jakkur P.O., Bengaluru 560064, Karnataka, India; 2 Division of Theoretical Chemistry and Biology, School of Biotechnology, KTH Royal Institute of Technology, S-106 91 Stockholm, Sweden

## Abstract

A major challenge in the Alzheimer’s disease (AD) is its timely diagnosis. Amyloid β (Aβ) aggregates have been proposed as the most viable biomarker for the diagnosis of AD. Here, we demonstrate hemicyanine-based benzothiazole-coumarin (**TC**) as a potential probe for the detection of highly toxic Aβ_42_ aggregates through switch-on, enhanced (~30 fold) red fluorescence (E_max_ = 654 nm) and characteristic colorimetric (light red to purple) optical outputs. Interestingly, **TC** exhibits selectivity towards Aβ_42_ fibrils compared to other abnormal protein aggregates. **TC** probe show nanomolar binding affinity (*K*_*a*_ = 1.72 × 10^7^ M^−1^) towards Aβ_42_ aggregates and also displace ThT bound to Aβ_42_ fibrils due to its high binding affinity. The Aβ_42_ fibril-specific red-shift in the absorption spectra of **TC** responsible for the observed colorimetric optical output has been attributed to micro-environment change around the probe from hydrophilic-like to hydrophobic-like nature. The binding site, binding energy and changes in optical properties observed for **TC** upon interaction with Aβ_42_ fibrils have been further validated by molecular docking and time dependent density functional theory studies.

The misfolding driven aggregation process of Aβ peptides in the brain is one of the main causes of Alzheimer’s disease (AD)^1^,^2^,^3^. The neurodegeneration and subsequent progressive deterioration in cognitive ability are hallmark symptoms of this incurable syndrome. The Aβ_42_ peptide with 42 amino acids has been shown to be highly susceptible to aggregation and toxic behavior among all the Aβ peptides (36–43)^3^,^4^,^5^. The aggregation process of Aβ peptides leads to formation of polymorphic oligomers, protofibrils, and fibrils which individually display a range of cellular toxicities^6^. Initially, Aβ fibrils were considered the neurotoxic form and causative agent of AD, whereas research in the last decade has revealed that oligomers are the most toxic form of Aβ causing oxidative stress, interacting with signaling receptors, disturbing metal homeostasis and disrupting neuronal cell membrane^3^,^7^. Membrane disruption is one of the major pathway of toxicity induced by Aβ oligomers^8^. Ramamoorthy *et al*. have recently shown that Aβ exhibit two-step mechanism for membrane disruption, i) Aβ interacts with gangliosides present on the cell membrane to form ion channel-like pores and ii) Aβ fibrillization itself induce membrane fragmentation of lipid bilayer^9^. Aβ_42_ aggregates are an attractive biomarker to target for diagnosis and therapeutics of AD. One of the major problems in the diagnosis of AD is the lack of effective methods for the selective detection of Aβ_42_ aggregates. While diagnosis of AD is traditionally based on behavioral tests or cognition in patients, several imaging technologies such as positron emission tomography (PET)^10^, magnetic resonance imaging (MRI)^11^, and single-photon emission computed tomography (SPECT)^12^ have been developed for the detection of Aβ_42_ aggregates. However, these technologies are still limited by several obstacles, like long data acquisition time, radioactive exposure, poor resolution and need of expensive equipment. Optical imaging using fluorescence and colorimetric probes has emerged as a potential alternative technique as it offers real-time, nonradioactive, high-resolution imaging for inexpensive diagnostics and screening of drugs for AD^13^,^14^,^15^. Thioflavin T (ThT) is the most extensively used fluorescence probe for the *in vitro* detection and staining of Aβ_42_ fibrillar aggregates however, it suffers from poor selectivity and often leads to false detection^16^,^17^. In the past few years, derivatives of oxazine^18^, BODIPY^19^, curcumin^20^, styryl^21^ fluorescein^22^ and benzothiazole^23^ have been developed and used as fluorescence probes for Aβ_42_ fibrillar aggregates, these probe lack selectivity for Aβ_42_ fibrils over other peptide/protein based aggregates.

An ideal fluorescence probe must exhibit certain characteristic properties to be used as a diagnostic probe for Aβ_42_ fibrillar aggregates in AD viz., i) high specificity and strong binding affinity, ii) emission in the optical window of 500–750 nm with a large Stokes shift, iii) switch-on fluorescence change upon binding with Aβ_42_ fibrillar aggregates, and iv) ability to rapidly cross the blood brain barrier (BBB). Further, mixed dementia is another state in which abnormal characteristics of more than one type of dementia occur simultaneously and in such cases, determining the specific type of neurodegenerative disorder in the patient is very crucial. Therefore, there is an urgent need for developing probes which could selectively differentiate toxic aggregates responsible for specific neurodegenerative disease. Unfortunately, there is lack of studies on probes that selectively differentiate plaques responsible for any specific disorder. We lack fluorescence probes which selectivity binds to specific aggregates, as most of them fluoresce upon binding to forced or artificially formed protein aggregates generally observed in all kinds of dementia^24^. Recently, J. Yang *et al*. reported an amino naphthalene 2-cyanoacrylate based fluorescence probe, which discriminates between Aβ and Prion plaques by means of differential mode of binding attributed to microenvironments in the binding pockets^25^. However, still there is a need for many more probes which can selectively differentiate other important neurodegenerative disorders. Oligomers being the most toxic form of Aβ causing neuronal death in AD, much efforts are devoted towards studying its structure and designing probes for detection of oligomers. Recently, Knowles *et al*. revealed that the formation of Aβ oligomers depends on the amount of both Aβ monomers and Aβ fibrils. Initially, Aβ aggregates formed through primary nucleation where Aβ monomers self-assemble to fibrils through oligomers as intermediate state. Once a certain concentration of Aβ fibrils is reached they act as secondary nucleation site and initiate the formation of Aβ oligomers from the monomers on their surface this phenomenon is called as secondary nucleation^26^. Therefore designing inhibitors and probes for both Aβ fibrils and oligomers are essential for treating AD and studying its progression^27^,^28^,^29^. Colorimetric detection of Aβ_42_ fibrillar aggregates using antibodies has been demonstrated, but this technique is complicated and expensive^30^. With this background, the need for developing selective fluorometric and colorimetric probes based on simple organic molecules which are easy to handle and offer quick detection is strongly indicated. In this context, we report a hemicyanine derivative as a high affinity, selective, switch-on red fluorescence and colorimetric probe **TC** for Aβ_42_ fibrillar aggregates. **TC** exhibits better detection properties over previously reported fluorescent probes (Table S1).

## Results and Discussion

**Figure 1:**
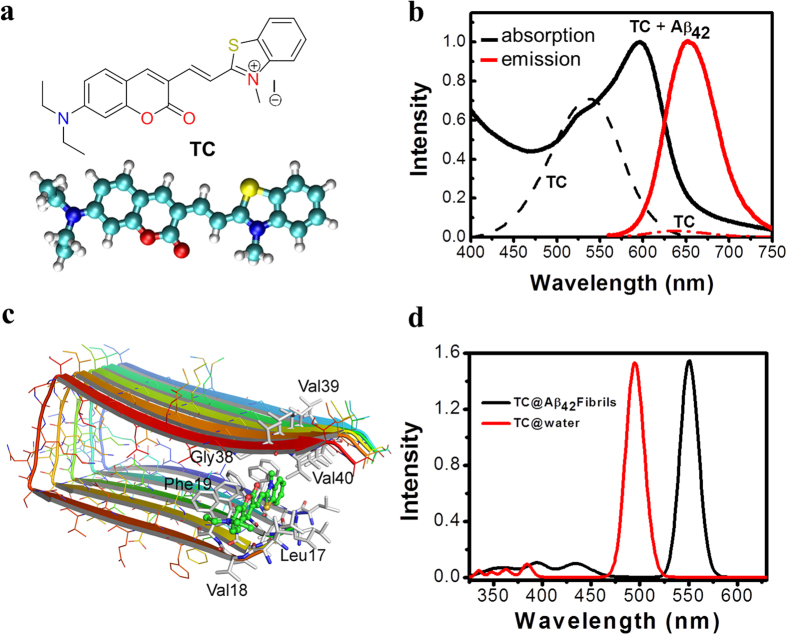
(**a**) Molecular and energy minimized structures of probe **TC**. (**b**) Absorption and emission (λ_ex_ = 537 nm) spectra of probe **TC** in presence and absence (doted lines) of Aβ_42_ fibrillar aggregates. (**c**) The binding mode of **TC** in the entry site of Aβ_42_ fibril. The Aβ_42_ fibril is shown in cartoon mode, the binding site residues in stick mode and **TC** in stick and ball mode (PyMol 1.3). (**d**) The absorption spectra computed for **TC**@water and **TC**@Aβ_42_ fibril system using TD-DFT/MM models.

ThT has been extensively used to stain Aβ_42_ fibrillar aggregates for the past few decades. This probe mainly consists of electron donating (N,N-dimethylaniline) and electron withdrawing (benzothiazole) moieties. The benzothiazole group is known to play a crucial role in the interaction of ThT on the surface of Aβ_42_ fibrillar aggregates^31^,^32^. The major drawbacks of ThT and many other probes involves lack of selectivity and low affinity, which encouraged us to search for a new, more effective fluorescence probe for Aβ_42_ fibrillar aggregates based on the benzothiazole platform, with high selectivity and affinity. We chose to investigate the hemicyanine-based (benzo)thiazole-coumarin (**TC**) conjugate as a ‘fluorescence-ready’ probe for Aβ_42_ fibrillar aggregates (Fig. 1). A benzothiazole conjugate with hydrophobic pyrene chromophore (**TP**) was also included in our studies (Fig. S1). These compounds are recently reported by our team and discovered **TC** as an effective switch-on red fluorescence probe for DNA containing AT sequences 33. To our surprise, **TC** with benzothiazole and coumarin moieties was found to exhibit highly enhanced fluorescence with superior selectivity and sensitivity for Aβ_42_ aggregates with higher affinity compared to DNA. Furthermore, the **TC** and **TP** probes with molecular weights in the optimum range of ~350–550 Da, and possesses appropriate log P values and number of hydrogen bond donors and acceptors (Fig. S1)^34^.

**Figure 2:**
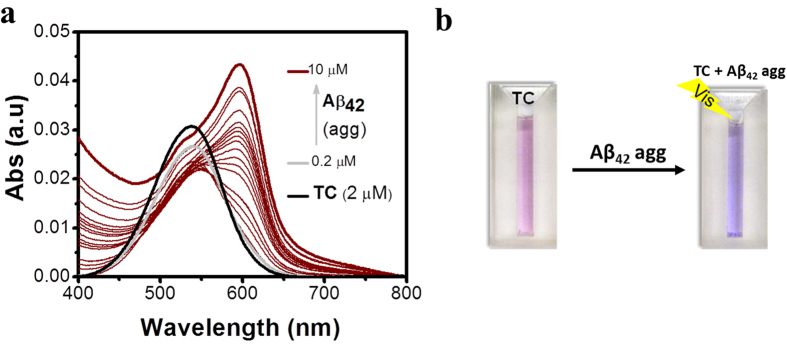
(**a**) Absorption (Abs) spectra of probe **TC** (2 μM) with increasing concentration of Aβ_42_ fibrils (0.2, 0.4, 0.6, 0.8, 1.0, 1.2, 1.4, 1.6, 1.8, 2.0, 2.5, 3.0, 3.5, 4.0, 4.5, 5.0, 6.0, 7.0 and 10.0 μM). (**b**) Photographs of **TC** (2 μM) and **TC** (2 μM) + Aβ_42_ fibrils (15 μM) showing a colorimetric change from pale pink to purple.

Initially we studied the molecular interactions of **TC** and **TP** in the absence and presence of Aβ_42_ aggregates through the absorption and emission measurements in PBS buffer (10 mM, pH = 7.4). Mature Aβ_42_ fibrillar aggregates were prepared following the procedure reported in the literature (Supplementary Information). **TC** and **TP** showed absorption bands at 537 nm and 460 nm, respectively, and very weak emissions at 638 nm and 623 nm, respectively, in the absence of Aβ_42_ fibrillar aggregates (Figs 1b and S1). In the presence of Aβ_42_ fibrillar aggregates (10 μM), **TC** (2 μM) showed a remarkable increase in the absorption maxima (hyperchromicity) with an enormous bathochromic shift (Δλ_max_ ≈ 59 nm) relating to solution color change from pale pink to purple (Fig. 2). To elucidate the observed spectral changes of **TC**, we carried out concentration-dependent studies of Aβ_42_ fibrillar aggregates against a fixed concentration of **TC** (2 μM). Initially, **TC** exhibited a decrease in absorption intensity in the concentration range 0–1 μM of Aβ_42_ fibrillar aggregates. In addition, a shoulder band was observed for 0.8 μM of Aβ_42_ at 595 nm. Further, with increasing concentration of Aβ_42_ fibrillar aggregates (1–10 μM) the shoulder band at 595 nm became more prominent with strong absorption (Fig. 2a). The bathochromic shift in the absorption band of **TC**, in the presence of Aβ_42_ fibrillar aggregates indicated their favorable interactions. The observed colorimetric change (pale pink to purple) as a consequence of binding of **TC** to Aβ_42_ fibrillar aggregates may be attributed to aggregate-induced changes in the intramolecular alignment and electronic structure of **TC** (Fig. 1)^35^,^36^. In similar absorption studies with Aβ_42_ fibrillar aggregates, **TP** failed to exhibit any detectable change in absorption and in the color of the solution.

In order to characterize the aggregate-specific shift in the absorption spectrum of **TC** and to propose its absorption maximum as a “colorimetric signature” for amyloidosis, its one photon absorption properties were computed by employing time-dependent density functional theory (at the B3LYP/TZVP level) in polar, non-polar and fibril-like environments. In particular static and dynamic results were presented where the former one involves a single optimized geometry of **TC** in the specific solvent environments while the latter results are obtained as average over numerous configurations from Car-Parrinello QM/MM molecular dynamics. These models are respectively referred to as TD-DFT/PCM and TD-DFT/MM^37^. For further details, we refer to the computational details section of supplementary Information. The calculation only for the most stable binding mode of **TC** in fibril as shown in Fig. 1c has been carried out. Representative snapshots used in TD-DFT/MM calculations for **TC**/fibril and **TC**/water systems are shown in Fig. S2. The spectra computed only for dynamic models (by convoluting the absorption bands of six lowest energy excitations) are shown in Fig. 1d. The absorption spectrum is characterized by a single dominant band in the visible region which is due to the lowest frequency excitation of π-π^*^ character. The molecular orbitals involved in this excitation are shown in the supplementary information (Fig. S3). The absorption maximum (λ_max_) for **TC** from the aforementioned models is listed in Table 1 along with the experimental results which show a red shift by 58 nm for the **TC** probe going into the fibril-like environment.

The simplistic polarizable continuum model reproduces the trend of a red-shift in the absorption spectra of **TC** when going from water-like to non-polar, chloroform environment even though the size of the shift is small (14 nm) when compared to experiment (24 nm). Based on this result, it can be suggested that the hydrophilic-like to hydrophobic-like change in the micro-environment may be a feasible mechanism for the fibril-induced red-shift in the absorption spectra of **TC**. The more sophisticated TD-DFT/MM approach which accounts for electrostatic and polarization interactions between **TC** and its fibril-like and aqueous environment also confirmed this and reproduce the red shift (56 nm) in excellent agreement with experiment. Usually, the change in the micro-environment alters the molecular structure and conformation of the probe which also significantly contributes to the shift in the spectra^38^. For this reason, we investigated the fibril-induced changes in conformation and molecular structure (along the conjugation pathway) of **TC** and interestingly, this does not change significantly (refer to section 3.0 of the supplementary Information) and so only contributes to the shift by 4 nm. The absolute λ_max_ is underestimated in our model which refers to limitations of the QM model itself. Our motivation though, is to explain the possible origin for the observed red shift due to change in environment (aqueous to fibril), something that is allowed by the excellent reproduction of the red shift by the more advanced TD-DFT/MM model. The characterization of the micro-environment of **TC** binding site clarifies its hydrophobic nature, and we can attribute the change in the hydrophilic-like to hydrophobic-like environment around **TC** when it binds to the fibril as the responsible factor for the red shift.

**Figure 3:**
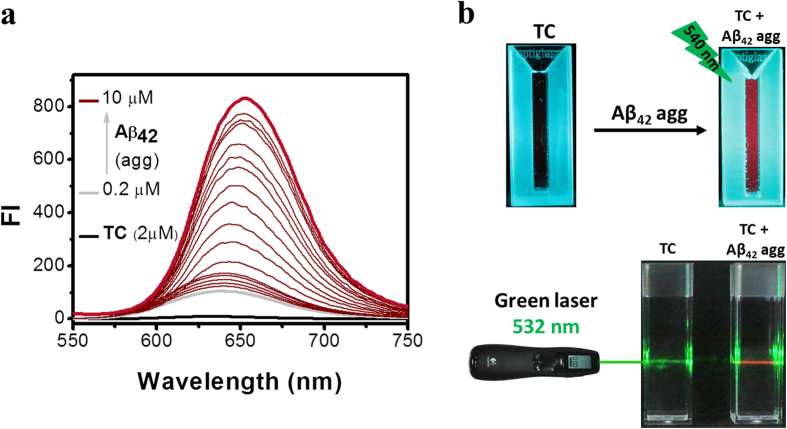
(**a**) Emission (FI) spectra (λ_ex_ = 537 nm) of probe **TC** (2 μM) with increasing concentration of Aβ_42_ fibrils (0.2, 0.4, 0.6, 0.8, 1.0, 1.2, 1.4, 1.6, 1.8, 2.0, 2.5, 3.0, 3.5, 4.0, 4.5, 5.0, 6.0, 7.0 and 10.0 μM). (**b**) Photographs of **TC** and **TC** (2 μM) + Aβ42 (15 μM) fibrils samples illuminated under green light (540 nm), **TC** (2 μM) + Aβ_42_ (15 μM) illuminated with laser emitting green light (532 nm) shows a red beam in the sample solution.

Subsequently, we performed fluorescence titration experiments to probe the response of **TC** in the presence of Aβ_42_ fibrillar aggregates. The emission spectrum of **TC** (2 μM) exhibited a ~30-fold fluorescence enhancement (E_max_ = 654 nm) when bound to Aβ_42_ fibrillar aggregates. The quantum yield of probe **TC** alone in PBS (10 mM) is 0.073, while **TC** bound to Aβ_42_ fibrillar aggregates showed appreciable quantum yield of 0.40 (Fig. 3). Again, **TP** did not show any detectable change in the fluorescence behavior in the presence of Aβ_42_ fibrillar aggregates. The switch-on red fluorescence of **TC** is a typical behavior of cyanine-based probes, which are known to form twisted intramolecular charge transfer (TICT) complexes in the excited state and exhibit fluorescence emission in response to a surrounding environment^39^. **TC** probe alone is non-fluorescence in buffer due to internal non-radiative molecular twisting and self-aggregation, whereas the intramolecular twisting is restricted upon binding to Aβ_42_ fibrillar aggregates leading to enhanced (~30-fold) red fluorescence^40^,^41^. Aβ_42_ fibrillar aggregates (30 μM) were incubated with ThT (5 μM) and **TC** (5 μM) for 5 min and imaged under fluorescence microscope. Characteristic Aβ_42_ fibrillar aggregates can be distinctly seen in TEM images, whereas fluorescence images show large clumps of Aβ_42_ aggregates owing to low resolution of the technique (Fig. S7). Probe **TC** did not show appreciable changes in the absorption and emission properties under different buffer conditions which indicate that solvent (buffer solution) has no significant effect on the conformational or aggregation tendency of the probe (Fig. S4). Furthermore, a pH-dependent study showed that photophysical properties of probe **TC** is not affected in the pH range of 3–8, which reaffirm the utility of the probe in most physiological conditions (Fig. S5).

**Figure 4:**
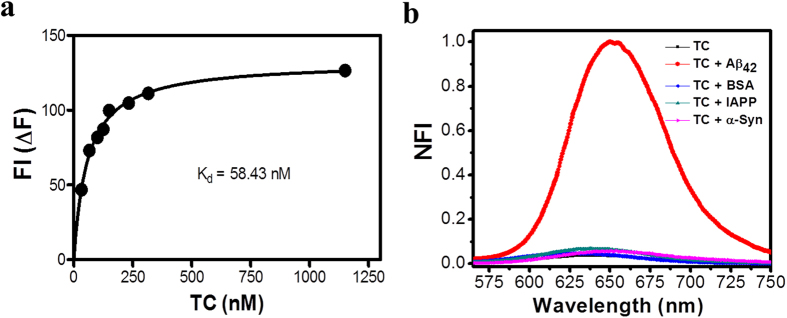
(**a**) Plot of the difference in fluorescence intensity (ΔF) as a function of the concentration of **TC** in the presence of Aβ_42_ fibrillar aggregates (2 μM) in solutions (10 mM PBS). (**b**) Normalized fluorescence intensity (NFI) of **TC** upon interaction with aggregates of Aβ_42_ (5 μM), α-synuclein (α-Syn) (20 μM), amylin (IAPP) (20 μM) and hydrophobic protein bovine serum albumin (BSA).

Next, we calculated the binding constant by studying the fluorescence response with varying concentration of **TC** against a fixed concentration of Aβ_42_ fibrillar aggregates (dose-dependent saturation assay, Fig. S8). The obtained standard saturation curve was fitted to a single-binding site, which gave a dissociation constant *K*_*d*_ of 58 ± 1.2 nM (the association constant was calculated to be *K*_*a*_ = 1.72 × 10^7 ^M^−1^ for 2 μM of Aβ_42_ fibrillar aggregates) (Fig. 4a). Notably, our recent study showed that AT-selective binding of **TC** to a DNA duplex generates a ~16-fold fluorescence enhancement and *K*_*d*_ in the micromolar range (10 μM)^33^. Remarkably, the current study reveals a ~30-fold fluorescence enhancement with *K*_*d*_ in the nanomolar range indicating a much higher binding affinity of **TC** towards Aβ_42_ fibrillar aggregates compared to DNA^42^. To further evaluate high affinity of **TC** towards Aβ_42_ fibrillar aggregates compared to DNA, we have performed a competitive binding experiment. The competitive binding experiment is based on the fact that, when **TC** binds to either Aβ_42_ fibrillar aggregates or DNA show characteristic changes in both absorption and emission spectra corresponding to probe **TC** and changes observed in each case are substantially different. First, probe **TC** was saturated with excess DNA (calf thymus) which showed characteristic changes in both absorption and emission spectra of **TC**, which corresponds to DNA binding, but when the same sample was added with Aβ_42_ fibrillar aggregates (10 μM, incubate for 15 min), it exhibited changes in both absorption and emission spectra which were similar to absorption and emission features corresponding to **TC** bound to Aβ_42_ fibrillar aggregates alone. This observation highlights the fact that in the presence of both Aβ_42_ fibrillar aggregates and DNA it preferably binds to Aβ_42_ fibrillar aggregates over DNA (Fig. S9). In addition, the *K*_*d*_ of Aβ_42_ fibrillar aggregates bound **TC** is very low compared to that of the control probes ThT (~0.8 μM) and Congo red (~1.1 μM) confirming the superiority of the **TC** probe in terms of binding affinity towards Aβ_42_ fibrillar aggregates^43^,^44^. Oligomers and fibrils are prominent polymorphic forms of Aβ_42_ aggregates. Probe **TC** showed selective fluorescence enhancement towards Aβ_42_ fibrils over oligomers which exhibited a slight red shift (8 nm) in the basal fluorescence and negligible fluorescence enhancement (Fig. S10). Further we have performed fluorescence studies in the presence of intracellular protein content bovine serum albumin (BSA), fibrillar aggregates of α-synuclein (α-Syn) and islet amyloid polypeptide (IAPP, amylin) implicated in Parkinsons disease and type II diabetes, respectively. Incubation of **TC** with BSA, α-Syn aggregates and IAPP aggregates (20 μM) did not lead to significant fluorescence enhancements confirming the preferential selectivity of the probe towards Aβ_42_ fibrillar aggregates over other proteins and peptide aggregates (Fig. 4b).

**Figure 5:**
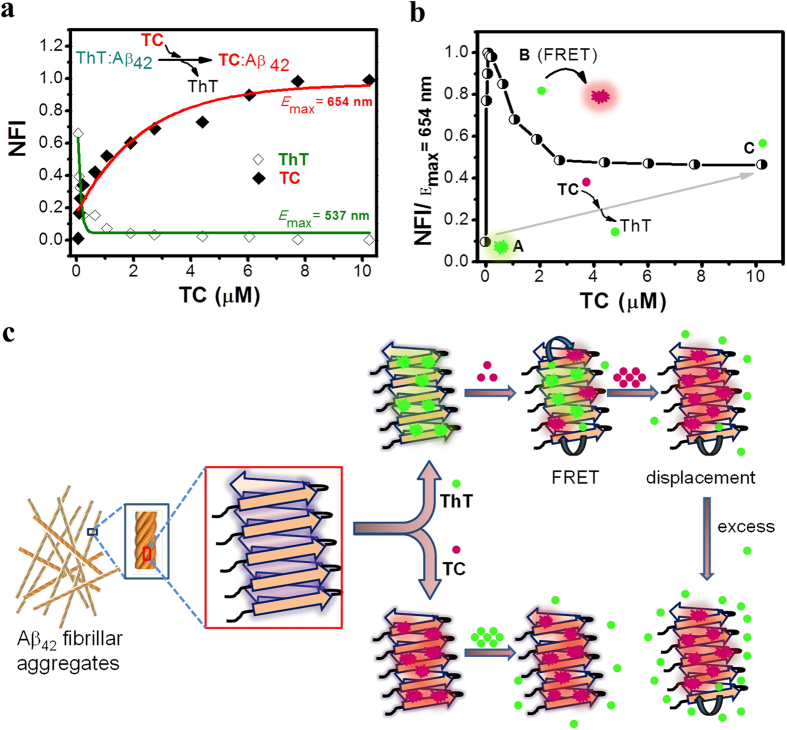
Displacement assay. (**a**) Titration of **TC** to ThT/Aβ_42_ fibrillar aggregate complex (ThT, 5 μM/Aβ_42_ fibrils, 10 μM) in 10 mM PBS buffer solution. High affinity **TC** effectively displaces ThT from the ThT/Aβ_42_ fibrillar aggregate complex, as monitored by the decrese in fluorescence emission at 483 nm (◊ green trace, λ_ex_ = 450 nm) and corresponding increase in fluorescence emission at 654 nm (♦ red trace, λ_ex_ = 537 nm). (**b**) In displacement assay (**a**) emission of **TC** monitored at 654 nm (*E*_max_) upon excitation at 450 nm (λ_ex_ of ThT). Region A: ThT/Aβ_42_ fibrillar aggregate complex. Region B: **TC**/ThT/Aβ_42_ fibrillar aggregate complex, at low concentration **TC** coexists with ThT leading FRET between them. Region C: **TC** displaces ThT, with residual ThT (possibaly in the inner cleft of the Aβ_42_ fibril) which leads to residual FRET. (**c**) Proposed model for the **TC** displacement of ThT and FRET between them on the Aβ_42_ fibrils. NFI: Normalized fluorescence intensity.

**Figure 6:**
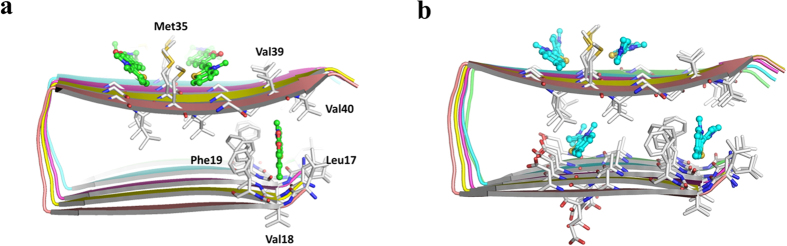
Docking results of (**a**) **TC** and (**b**) ThT with Aβ_42_ fibril (all binding sites are shown). The fibril is shown in cartoon mode, the binding site residues in stick mode and **TC** or ThT in stick and ball mode.

Recently, Suzuki *et al*. have studied the competitive binding of Aβ inhibitor, EGCG and ThT towards Aβ aggregates using ^19^F NMR to understand its binding interactions^45^. Similarly, to gain further insight into the binding interaction of **TC** probe, displacement assay was performed against ThT-bound Aβ_42_ fibrillar aggregates. The well-separated emission spectra of ThT (green region) and **TC** (red region) made it possible to observe fluorescence changes corresponding to individual probes during the displacement experiments (Fig. S12). Remarkably, a gradual addition of **TC** to the ThT/ Aβ_42_ fibrillar aggregate complex (ThT = 5 μM and Aβ_42_ = 10 μM) resulted in a steady decay in fluorescence at 483 nm (λ_ex_ = 450 nm) and a corresponding enhancement in the emission intensity at 654 nm (λ_ex_ = 537 nm). This clearly suggested an effective displacement of ThT by **TC** owing to the formation of a much stronger **TC**/Aβ_42_ fibrillar aggregate complex (Fig. 5a). An interesting observation was made during the titration studies where spectral features corresponding to emission of **TC** (at 654 nm) were observed upon excitation of the sample (**TC**/ThT/Aβ_42_ fibrillar aggregates) at 450 nm (ThT excitation wavelength). Addition of **TC (**33 nM to 10.233 μM) to the ThT/ Aβ_42_ fibrillar aggregate complex showed a gradual decrease in the fluorescence emission at 483 nm (ThT) as expected. However, upon 450 nm (ThT) excitation, fluorescence was also observed at 654 nm (**TC)** with a slight red shift. The fluorescence intensity of this unprecedented emission band (**TC**) decreased slowly with further increase in the concentration of added **TC** and finally reached a constant value (Fig. 5b). These changes in the emission characteristics, particularly the fluorescence emission of **TC** upon excitation corresponding to ThT is attributed to fluorescence resonance energy transfer (FRET) between the Aβ_42_ fibrillar aggregates bound to ThT and **TC**. Evidently, the emission spectrum of ThT significantly overlaps with the absorption spectrum of **TC** making them a suitable donor-acceptor pair on the aggregate surface (Fig. S12)^46^,^47^. At the beginning of the titration, **TC** binds to the ThT/Aβ_42_ fibrillar aggregates complex by partially displacing ThT, leading to FRET between bound ThT (donor) and **TC** (acceptor) (Fig. 5b). For concentrations of **TC **> 150 nM, displacement of ThT by **TC** resulted in a decreased FRET-fluorescence of **TC** (Fig. S6). However, the FRET-based fluorescence at 654 nm was not quenched completely due to persistent residual ThT-**TC** pairs on an Aβ_42_ fibrillar aggregates. The quenching of fluorescence intensity of ThT (at 483 nm) to its basal level indicates that **TC** binds to similar primary binding pockets of Aβ_42_ fibrillar aggregates occupied by ThT. On the other hand, excitation at 537 nm (**TC**) showed a gradual increase in fluorescence independent of ThT displacement, confirming the presence of multiple binding sites for **TC** on Aβ_42_ fibrillar aggregates (Fig. 6). The displacement of ThT was almost instantaneous and did not require any incubation time. Addition of **TC** (1 μM) to the ThT (10 μM)/Aβ_42_ (50 μM) complex led to a complete change in emission color of the sample, from green to bright pinkish red, as seen under UV-light illumination (λ_ex_ = 365 nm). Addition of excess ThT (50 μM) did not displace **TC** from its complex with Aβ_42_ fibrillar aggregates owing to the high binding constant (Fig. S13). The Aβ_42_ fibrillar aggregates stained with **TC** retained red fluorescence even after 50 days of aging, thus further indicating the strong binding affinity and non-dissociative nature of **TC** upon binding to Aβ_42_ fibrillar aggregates.

In order to get a microscopic picture of the **TC** to fibril binding, we carried out a molecular docking study. In agreement with experimental indications, our study shows that there are multiple binding sites (such as entry cleft and surface) in the fibril accessible for binding of **TC** (Fig. 6). However, the most favorable binding site was identified in the entry site formed by Leu17, Val18, Phe19, Gly38, Val39 and Val40 (Fig. 1c). The binding affinity calculated by AutoDock is the highest in this site (about −8.5 kcal/mol), whereas in other sites, it is in the range of −6.0 ~ −8.0 kcal/mol. A flexible molecular model for **TC** during the docking yields a binding affinity equivalent to −9.86 kcal/mol which corresponds to *K*_*d*_ = 55.5 nM (which is in good agreement with experimental data). As **TC** is positively charged, it is unfavorable to bind in the inner sites which are fully buried, and the partially buried entry site is more favorable. Figure 1c shows that **TC** is clamped in the entry site mainly through hydrophobic interaction with Leu17 and Val39 through π-π stacking interaction with the phenyl ring of Phe19. Water molecules can also enter this site to solvate the positive charge of **TC**. It is relevant to note that all the amino acids form this binding pocket are hydrophobic and hence we believe that the red shift in the spectra is due to the change from hydrophilic to hydrophobic like micro-environment around the probe. Further, the bulky nature of the diethyl amino group makes it impossible for the **TC** probe to become buried inside the binding site, rather it is partly exposed to the solvent environment (Fig. S3a). Figure 6a,b show all possible binding sites available for **TC** and ThT in the fibril. The **TC** binds to the entry cleft, inner core and surface binding sites while **TC** binds only to the entry cleft and the surface binding sites which have to be attributed to the larger van der Waals surface associated with the latter molecule. Due to the larger binding affinity of **TC** towards the amyloid peptide, it can replace the ThTs in the entry cleft and other surface binding sites (which is supported by FRET data). However, ThTs in the core sites cannot be displaced by **TC**s and these therefore contribute to the population of residual that-**TC** pairs on Aβ_42_ aggregate contributing to the significant FRET intensity as discussed above. Additionally, we performed docking studies of **TC** with α-synuclein (PDB code: 4R0U) and IAPP (PDB code: 2KIB) fibrils^48^,^49^. It is found that **TC** can only be docked to the surface or the flanks of these two fibrils. It cannot be docked into the core sites (in particular to entry cleft site) as in the case of the Aβ_42_ fibril. The docking scores (empirical binding free energies) of **TC** with α-synuclein and IAPP are in the range between −5.0 ~ −7.0 kcal/mol, which are much lower (in terms of magnitude) than that with the Aβ_42_ fibril in the entry site (−8.5 kcal/mol). This result suggests that **TC** binds much more favorably with Aβ_42_ fibril than with α-synuclein and IAPP (Fig. S14).

## Conclusion

We demonstrated that the hemicyanine-based benzothiazole-coumarin (**TC**) probe binds to Aβ_42_ aggregates with nanomolar affinity (K_a_ = 1.72 × 10^7 ^M^−1^). The probe showed switch-on red fluorescence with a large Stokes shift (~117 nm) upon binding to Aβ_42_ aggregates along with a characteristic colorimetric response which can be attributed to a change in the dielectric nature of the micro-environment around **TC** from hydrophilic-like to hydrophobic-like. The **TC** probe also showed good specificity as it did not interact with other abnormal protein aggregates of α-Syn and IAPP. Owing to high binding affinity, the **TC** probe displaced the ThT probe bound to Aβ_42_ aggregates, conversely very high concentrations of ThT could not displace **TC** bound to Aβ_42_ aggregates. The binding site in the Aβ_42_ fibril for **TC** has been revealed from molecular docking studies. We propose that optimization of **TC** as a lead probe for Aβ_42_ aggregates may afford novel, useful optical-based diagnostic probe for Alzheimer’s disease.

## Methods

All reagents and solvents were obtained from Sigma-Aldrich and used without further purification. All air and moisture sensitive reactions were carried out under an argon atmosphere. Absorption spectra were recorded with Perkin Elmer Model Lambda 900 spectrophotometer. Fluorescence spectral measurements were carried out by using Perkin Elmer Model LS 55 fluorescence spectrophotometer. Incubation for fibril formation was performed in the Eppendorf Inova42 incubator.

### Synthesis of probes

Probes **TC** and **TP** were synthesized following the literature procedure recently reported from our group^33^.

### Preparation of Aβ_42_ fibrillar aggregates^50^

Aβ_42_ peptide (0.25 mg) (Merck, calbiochem) was dissolved in hexafluoro-2-propanol (HFIP, 0.2 mL) and incubated at room temperature for 1 h. HFIP was then removed by a flow of nitrogen and further dried by vacuum. HFIP-treated Aβ_42_ was then dissolved in DMSO to a final concentration of 1 mM and diluted to 200 μM with 10 mM PBS buffer (pH 7.4). The solution was incubated at 37 °C for 48 h with gentle and constant shaking. The formation of Aβ_42_ fibrillar aggregates was confirmed by ThT assay, CD measurements and TEM (Fig. S11).

### Preparation of amylin (IAPP) fibrillar aggregates and α-Synuclein fibrils^51^,^52^

Amylin peptide (0.1 mg) (Merck, calbiochem) sample of was dissolved in 100 μL of acetonitrile to disrupt any pre-existing aggregates, and taken up in 200 μL of 10 mM PBS buffer (pH 7.4). The final concentration of acetonitrile in the fibrillization buffer was 10% (v/v). The solution was sonicated continuously for 1 min to break up any potential aggregates. To form fibrils, the sample was incubated at 37 °C without agitation in an eppendorf tube for 120 h (5 days). α-Synuclein peptide (0.5 mg) (Sigma-Aldrich) was dissolved in hexafluoro-2-propanol (HFIP, 0.2 mL) and incubated at room temperature for 1 h. HFIP was then removed by a flow of nitrogen and further dried by vacuum. Then α-Synuclein peptide is dissolved TBS buffer to a concentration of 200 μM. Then the solution is incubated at 37 °C for 3–5 days with constant shaking of 150 rpm.

### Determination of the binding constant of TC for Aβ_42_ aggregates^19^

Increasing concentration of probe **TC** (0–1.15 μM) was titrated against a fixed concentration of Aβ_42_ aggregates (2 μM) and fluorescence intensity at 639 nm was recorded (λ_ex_ = 537 nm). The K_d_ binding curve was generated by GraphPad Prism 5.0 (GraphPad Software, Inc., La Jolla, CA, USA) by using below equation, where X is concentration of probe **TC** and Y is change in fluorescence intensity


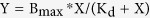


B_max_ is the maximum specific binding has the same units as Y.

K_d_ is the equilibrium binding constant.

### Molecular Docking

Molecular docking was performed using AutoDock 4.2, and the AutoDock-Tools software was used to set up the necessary inputs for the docking program^53^. The structure of fibril consisting of 5 Aβ_42_ peptides (PDB code 2BEG)^54^ was taken from the Protein Data Bank and was used as the protein model for docking in this study. The geometry of **TC** in gas phase was optimized at the level of B3LYP/6–31+G* using the Gaussian09 software. A grid box centered on the protein was defined with a dimension of 90 × 70 × 60 Å using a 0.375 Å grid step, which is large enough to encompass the whole protein and leave enough space for docking ligand on the surface. The Lamarckian Genetic Algorithm was used for legend conformation, search and was run for 100 times, which would generate 100 possible protein-ligand complexes. All other parameters were left as default. The resulting ligand conformers were clustered by root mean square deviation (RMSD).

## Additional Information

**How to cite this article**: Rajasekhar, K. *et al*. A High Affinity Red Fluorescence and Colorimetric Probe for Amyloid β Aggregates. *Sci. Rep.* **6**, 23668; doi: 10.1038/srep23668 (2016).

## Supplementary Material

Supplementary Information

## Figures and Tables

**Table 1:** The absorption maximum in nm (with oscillator strength shown in parenthesis) for TC in different micro-environment as predicted from TD-DFT/PCM, and TD-DFT/MM approaches.

Method	TC/chloroform	TC/fibril	TC/water	Shift, nm
**Static (TD-DFT/PCM)**	528(1.9)	–	514 (1.8)	14
**Dynamic (TD-DFT/MM)**	–	551 (1.6)	495 (1.5)	56
**Experiment**	561	595	537	58
