# Supplementary material for: A High Affinity Red Fluorescence and Colorimetric Probe for Amyloid β Aggregates
Source: Sci Rep. 2016 Apr 1;6:23668. doi: 10.1038/srep23668 (PMC4817056; doi:10.1038/srep23668)
Supplement: Supplementary Information [file srep23668-s1.pdf]

Supplementary Information

A High Affinity Red Fluorescence and Colorimetric Probe for Amyloid β Aggregates

K. Rajasekhar[1], *Nagarjun Narayanaswamy*[1], *N. Arul Murugan*[2], *Guanglin Kuang*[2], *Hans Ågren*[2], *and T. Govindaraju*[1*]

[1]Bioorganic Chemistry Laboratory, New Chemistry Unit,

Jawaharlal Nehru Centre for Advanced Scientific Research (JNCASR),

Jakkur P.O., Bangalore 560064, Karnataka, India

[2]Division of Theoretical Chemistry and Biology,

School of Biotechnology, KTH Royal Institute of Technology,

S-106 91 Stockholm, Sweden

Corresponding Author

* T. Govindaraju; Email: tgraju@jncasr.ac.in; Fax: (+) 91 80 2208 2627

Table of Contents

1. Methods

1.1. Calculation of partition coefficient (log P)

1.2. Quantum yields determination

1.3. Preparation of Aβ_{42} oligomeric aggregates

1.4. Dot Blot Analysis

1.5. Temperature dependent study

1.6. Fluorescence microscopic imaging of Aβ42 fibrillar aggregates

2. Computational details

2.1. Modeling the Aβ_{42} fibril-specific optical properties of **TC**

3. Computational results

3.1. Aβ_{42} fibril-induced structural changes in **TC**

4.1. pH dependent study

2. Table S1. Comparision of fluorescent probes

3. Figure S1. Molecular structures of **TC** and **TP** with their absorption and emission maxima

4. Figure S2. The representative snapshot configurations used in TD-DFT/MM calculation

5. Figure S3. The HOMO and LUMO of **TC** involved in the lowest energy excitation

6. Figure S4. Effect of buffers on photophysical properties of probe **TC**.

7. Figure S5. pH dependent study for probe **TC**

8. Figure S6. Effect of temperature on photophysical properties of probe **TC**

9. Figure S7. Fluorescence images of Aβ_{42} fibrillar aggregates

10. Figure S8. Binding constant of **TC** towards Aβ_{42} fibrillar Aggregates

11. Figure S9. Competitive binding assay.

12. Figure S10. Probe **TC** specificity towards Aβ_{42} fibrillar aggregates

13. Figure S11. TEM and dot blot analysis

14. Figure S12. FRET and displacement assay

15. Figure S13. Photographs of ThT displacement by **TC**

16. Figure S14. Docking mode of **TC** with α-synuclein and IAPP

10. REFERENCES

I. METHODS

1.1 Calculation of partition coefficient (log P). Log P value was calculated by Shake flask (or tube) method.[1] To an equilibrated solution of n-octanol and water in a separating funnel probe **TC** was added and agitated well. The solution was left for 30 min for proper separation of immiscible solvents and then the distribution of **TC** was calculated using UV spectroscopy and the obtained concentrations were used in following equation to obtain log P value

$$\log P = \log [\text{conc. of } \mathbf{TC} \text{ in n-octanol/conc. of } \mathbf{TC} \text{ in water}]$$

1.2 Quantum yields determination.[2] Fluorescence spectra were measured in 1 cm quartz cuvettes with spectroscopic grade solvents. Cresyl violet perchlorate in ethanol (ϕ = 0.54) was used as the standard for the fluorescence quantum yield calculation using the absorption of the test sample. The emission spectral area was obtained from 550-800 nm. Dilute solutions (10^{-6} M) were used to minimize reabsorption effects. Fluorescence measurement were made three times for each dye and averaged. Quantum yield was determined using the following equation:

$$\phi_{TC} = \phi_{stand} (F_{TC}/F_{stand}) \times (A_{stand}/A_{TC}) \times (n_{TC}^2/n_{stand}^2)$$

ϕ = Quantum yield, **F** = Area under fluorescence spectra, **A** = Absorption maxima, and **n** = Refractive index

1.3 Preparation of $A\beta_{42}$ oligomeric aggregates. $A\beta_{42}$ peptide (0.25 mg) (Merck, calbiochem) was dissolved in hexafluoro-2-propanol (HFIP, 0.2 mL) and incubated at room temperature for 1 h. HFIP was then removed by a flow of nitrogen and further dried by vacuum. HFIP-treated $A\beta_{42}$ was then dissolved in DMSO to a final concentration of 1 mM and diluted to 200 μM with 10 mM PBS buffer (pH 7.4). The solution was incubated at 37°C for 1 h with gentle and constant shaking and then the sample was incubated for 24 h at 4°C. The obtained sample was centrifuged

to pellet out higher order aggregates and the supernatant was used for further experiments. The formation of oligomers was confirmed by TEM and dot blot analysis (Figure 11).

1.4 Dot Blot Analysis. Samples were spotted on the PVDF membrane, non-specific sites were blocked by soaking in PBS and skim milk (5% BSA, 0.5-1 h) at room temperature. The membrane was incubated with primary antibody A11 (1:3000) (specific for oligomers) at 4 °C for overnight and washed with PBS (3 x 5 min). The membrane was then incubated with anti-mouse secondary antibody (1:10000) conjugated with HRP for 30 min at RT and washed with PBS (3 x 5 min). Finally, the membrane was incubated with ECL reagent for 1 min and the chemiluminescence was recorded using Biorad Chemidoc touch (Figure 11).

1.5 Temperature dependent study on probe TC. Temperature-dependent absorption and emission studies were performed for probe **TC**. Absorption (537 nm) and emission (638 nm) of **TC** (4 μM) was recorded at various temperatures under different buffer conditions. Probe **TC** in PBS showed minimal change in absorption spectra up to 70 °C, after which decrease in absorption was observed. Similar effect on the absorption behaviour of **TC** was observed as a function of temperature in Tris buffer. Whereas in HEPES buffer, there was no change in the absorption spectra up to 50 °C, after which increase in absorption was observed. The temperature-dependent fluorescence emission study of probe **TC** showed decrease in fluorescence irrespective of buffer conditions (Figure S6).

1.6 Fluorescence microscopic imaging of $A\beta_{42}$ fibrillar aggregates. $A\beta_{42}$ fibrillar aggregates (30 μM) were incubated with ThT (5 μM) and **TC** (5 μM) for 5 min. and imaged under fluorescence microscope. Characteristic $A\beta_{42}$ fibrillar aggregates were observed in TEM images,

whereas fluorescence images showed fibrillar aggregates as large clumps owing to lower resolution of the fluorescence microscope (Figure S7).

2 Binding mode of TC on Aβ_{42} fibril

2.1 Fibril-specific optical properties of TC:

To model the optical property of **TC** in water and in fibril environments, we have employed the popular integrated approach that has been successfully used to study many organic molecules in their bio-structure bound state or in solvents.[3-7] We employ molecular dynamics, Car-Parrinello QM/MM molecular Dynamics and TD-DFT/MM response in sequential way for the **TC** in water (referred as **TC**@water) and **TC** bound to fibril in water (**TC**@fibril) systems. The initial configuration for **TC**@fibril is based on the most stable **TC**-fibril complex as obtained from the molecular docking mentioned above (refer to Figure S9). The long time scale processes are captured by the former approach while the short time scale molecular vibrations of **TC** and environment-induced structural changes in **TC** are correctly reproduced by CP QM/MM MD approach. Finally, the one photon absorption properties are obtained from TD-DFT/MM approach. In particular, the excitation energies are obtained as the poles of the linear response function while the oscillator strengths are obtained from the first residue. In the molecular dynamics run, the charges for **TC** has been obtained using CHELP protocol as implemented in gaussian09 at B3LYP/6-31+G* level.[8] The force-fields used for **TC**, fibril and water are respectively GAFF, ff09SB and TIP3P. During the simulation, the protein peptide atoms were kept fixed with a weak restraint while the protein backbone atoms were allowed to move freely. The molecular dynamics run involved equilibration and production runs and during the entire run the **TC** molecule has been observed to be in the fibril-bound state. The simulation has been

carried out in isothermal-isobaric ensemble. The total time scale for the production run was around 20 ns. Similarly, separate MD simulations have been carried out for **TC** alone in water (and with a neutralizing chloride ion). The final configuration from these runs was used as input configuration for the subsequent Car-Parrinello QM/MM molecular dynamics. In this set of calculations, only the **TC** probe has been described using density functional level of theory while the environments were described using molecular mechanics force-fields as used in previous MD runs. In particular, the BLYP exchange correlation functional has been employed in this approach and the wavefunction is described using planewave basis set. Further to reduce the demanding computational time, only the valence electrons are described explicitly and the interactions between the core electrons and nucleus are described using pseudopotential. A time step of 2 au has been used to integrate Car-Parrinello equation of motion and the total time scale for the production runs are around 30 ps. The instantaneous configurations from the simulations corresponding to **TC**@water and **TC**@fibril systems were stored for the analysis of the structure and for the computation of one photon absorption properties. Using these snapshots two sets of time dependent density functional theory calculations referred as TD-DFT/MM-0 and TD-DFT/MM were carried out using Dalton software.[8] In particular, B3LYP exchange correlation functional in combination with TZVP basis set has been employed. The use of such basis set for the excited state calculations has been validated for a number of organic molecules in our previous studies. The use of B3LYP has also been validated through the calculation of the so-called spatial overlap diagnostic parameter, Λ (the value was around 0.6).[9-10] The benchmarking studies carried out by Peach *et. al* suggest the excitation energies calculated using B3LYP functional for the cases with $\Lambda<0.3$ are associated with larger errors which is not true the current case.[9-12] In the former set (TD-DFT/MM- 0), only the coordinates of **TC** obtained from ab initio

MD were used while in the latter case (TD-DFT/MM) the coordinates of the environment (i.e. water and fibril) were also used. In this set of calculations only the electrostatic interaction between the probe and its environment was accounted for and the charges as used were based on the molecular mechanics force-field as in MD. One can also employ a polarizable force-field for the environment by placing the polarizabilities on the atomic sites of fibril and water. The unavailability of polarizable force-fields for fibrils and the tedious and time taking derivation of this stopped us to pursue with this. The TD-DFT/PCM calculations for **TC** in chloroform and water solvents were carried out on the optimized geometries of **TC** in respective solvents. The size of the cavity for the solute is based on the default values in the PCM implementation in gaussian09 software. These calculations were carried out to provide first hand information about the origin for the fibril-induced red shift in the absorption spectra of **TC**.

3.1 Fibril-induced structural changes in TC:

Many of the probe molecules are known to display structural changes (either with respect to bond lengths along the conjugation pathway or of conformational nature) specific to local micro-environment. Usually such structural changes also contribute significantly to the change in one and two photon optical properties. To characterize any such contribution in the case of **TC**, we have also analysed the bond lengths along the conjugation pathway (using the ab initio MD trajectory corresponding to **TC**@water and **TC**@fibril systems) and the bond length values are reported below. As can be seen the change in bond lengths due to change in micro-environment is not very significant. We have also not found any significant change in the conformational nature and relative orientation of the two aromatic moieties.

System	N-C1	C1-C2	C2-C3	C3-C4	C4-C5	C=O	C-O
**TC**@water	1.36	1.43	1.37	1.44	1.46	1.23	1.40
**TC**@fibril	1.37	1.42	1.38	1.43	1.49	1.21	1.40

4. pH dependent study

We performed a pH-dependent study to explore the involvement of acid-base interactions between **TC** and the binding pockets of the Aβ_{42} fibrillar aggregates (Figure S5). The absorbance and fluorescence spectra at different pH values revealed that the colorimetric and emission properties of **TC** were unaffected in broader pH range of 3 to 8. This study revealed that the binding of **TC** to Aβ_{42} aggregates does not occur through acid-base interactions, but rather involve hydrophobic and other noncovalent interactions.

Table S1. Comparison table for fluorescent probes for Aβ aggregates

Probe	Detection mode	λ_{em}	Stroke shift	K_d	Specificity	Binding mode
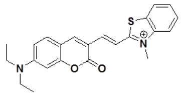 **TC, M.wt**= 391.94	**Fluorometric and colorimetric**	654 nm	117 nm	58 nM	Specific to Aβ aggregates over other peptide/protein aggregates	TC show hydrophobic interaction with Leu17 and Val39 and π-π stacking interaction with the phenyl ring of Phe19
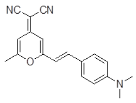 **PAD 1, M.wt**= 303.13	**Fluorometric**	570 nm	80 nm	58.9 nM	Not compared with other peptide/protein aggregates	Not defined
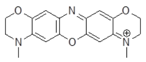 **AO1987, M.wt**= 324.13	**Fluorometric**	708 nm	55 nm	0.2 μM	Not compared with other peptide/protein aggregates	Not defined
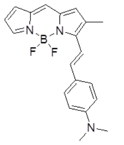 **BAP-1, M.wt**= 351.17	**Fluorometric**	648 nm	34 nm	44 nM	Not compared with other peptide/protein aggregates	Not defined
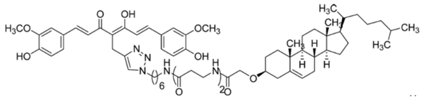 **BMAOI 14**	**Fluorometric**	484 nm	95 nm	0.8 μM	Not compared with other peptide/protein aggregates	Not defined
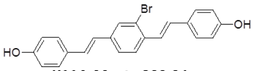 **K114, M.wt**= 392.04	**Fluorometric**	520 nm	45 nm	0.8 μM	Binds to Aβ peptide, α-synuclein, tau and other aggregates	Not defined
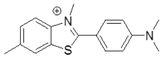 **ThT, M.wt**= 283.41	**Fluorometric**	485 nm	70 nm	25 nM	Binds to Aβ peptide, α-synuclein, tau and other aggregates	Have multiple binding sites
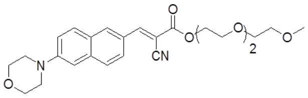 **ANCA-11, M.wt**= 410.46	**Fluorometric**	525 nm	95 nm	13.8 μM	Not compared with other peptide/protein aggregates	Not defined
**$[Ru(bpy)_2dppz]^{2+}$**	**Photoluminescence**			2.1 μM	Not compared with other peptide/protein aggregates	Val18 & Phe20

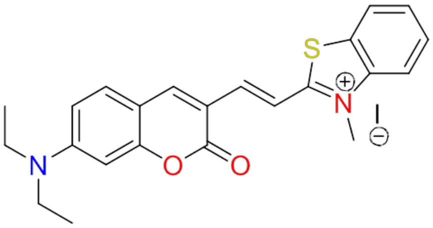

TC

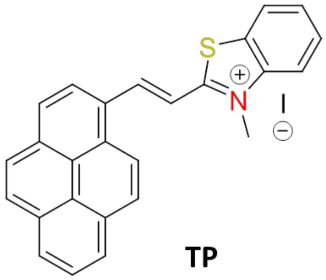

TP

Probe	Absorption maxima		Emission maxima		Molecular weight	logP	Hydrogen bond donor	Hydrogen bond acceptor
	without $A\beta_{42}$	with $A\beta_{42}$	without $A\beta_{42}$	with $A\beta_{42}$				
**TC**	537 nm	595 nm	638 nm	654 nm	391.14	3.2	3	1
**TP**	460 nm	462 nm	623 nm	626 nm	376.11	6.0	1	0

Figure S1. Molecular structures of probes **TC** and **TP** with their corresponding absorption and emission maxima in presence and absence $A\beta_{42}$ aggregates, and other properties.

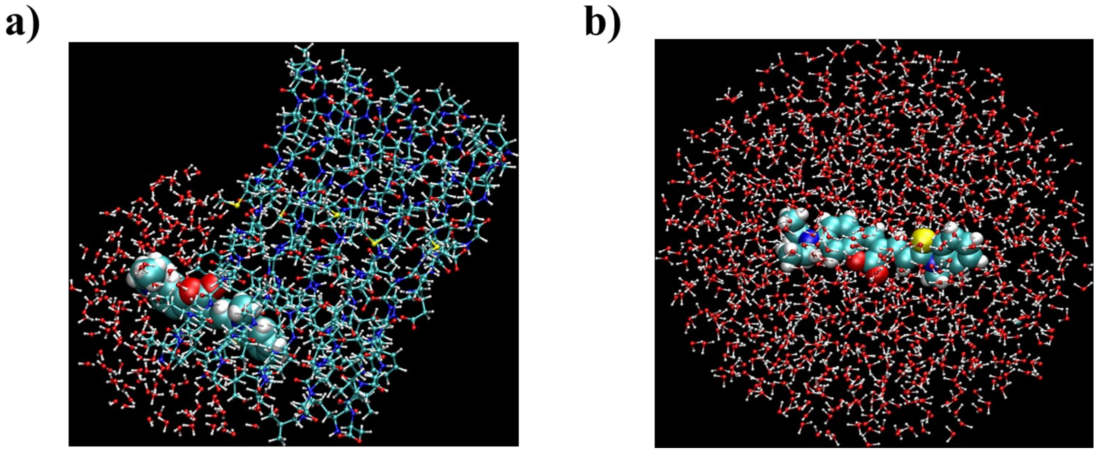

Figure S2. The representative snapshot configurations used in TD-DFT/MM calculation of a) **TC** in $A\beta_{42}$ fibrils and b) **TC** in water. In both cases **TC** is described using density functional theory (DFT) using B3LYP functional and TZVP basis set. The environments namely $A\beta_{42}$ fibrils in water and water solvents are described using molecular mechanics force-fields.

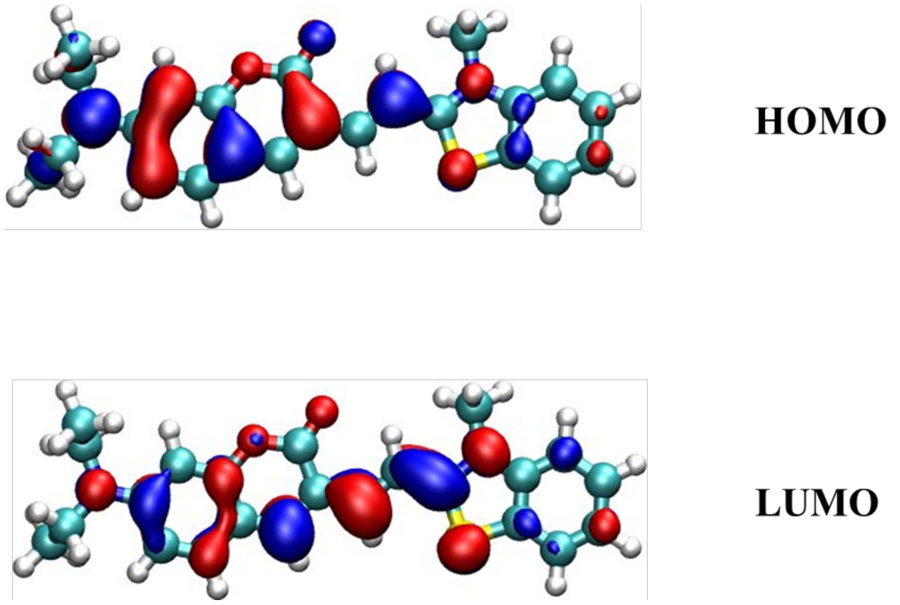

Figure S3. The HOMO and LUMO molecular orbitals of **TC** involved in the lowest energy excitation.

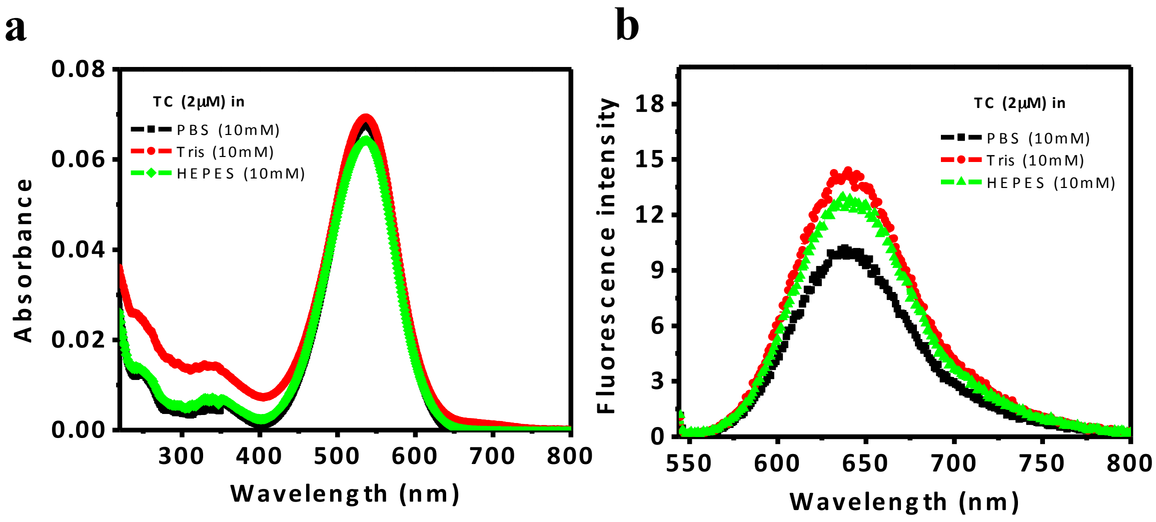

Figure S4. Effect of buffers (solvents) on photophysical properties of probe **TC**. a) absorbance and b) fluorescence (λ_{ex}= 537 nm) spectra of probe **TC** (1μM) in PBS (10 mM), Tris (10 mM) and HEPES (10 mM) buffer.

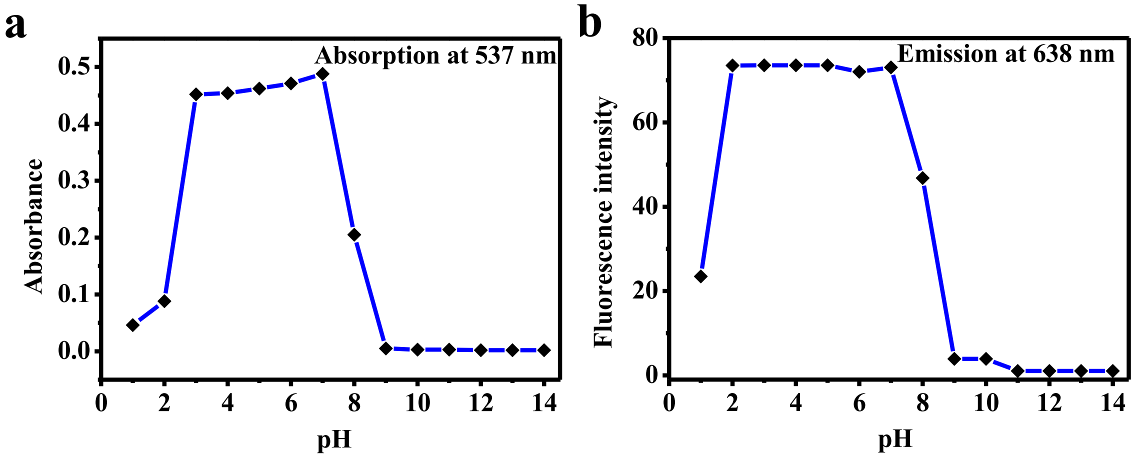

Figure S5. pH dependent study of probe **TC**. a) Absorbance (537 nm) and b) fluorescence (639 nm) of probe **TC** (10 μM) at different pH was recorded. From pH 3- 8 (which covers the physiological pH) the absorption and emission maxima remained unaffected indicating that probe **TC** is stable in physiologically relevant pH, whereas at pH 1 and pH 9 - 14 there was a sharp decrease in both emission and absorption maxima.

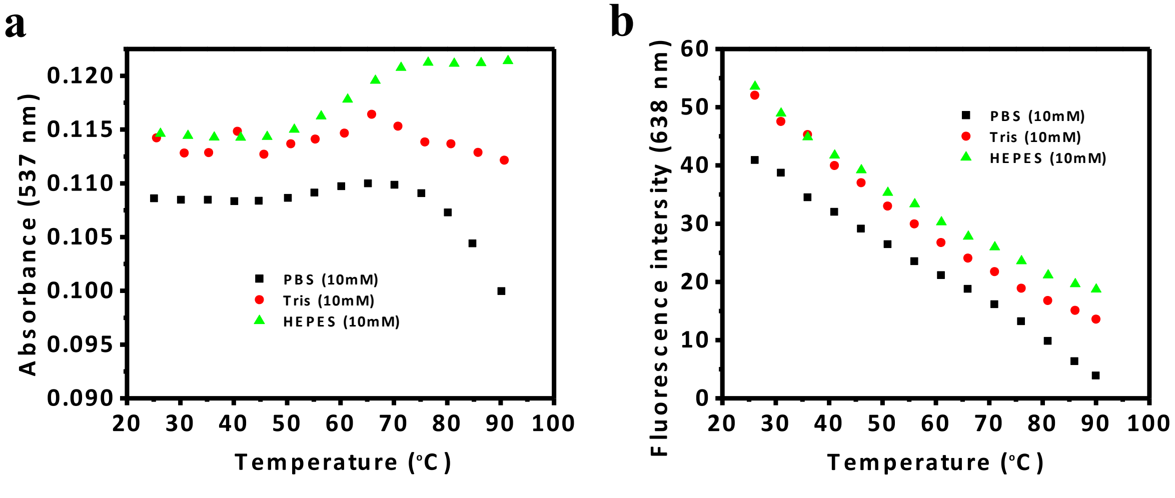

Figure S6. Effect of temperature on photophysical properties of probe **TC** (4 μM) in different buffers. a) absorbance at 537 nm and b) fluorescence (λ_{ex}= 537 nm) at 638 nm was recorded for probe **TC** in PBS (10 mM), Tris (10 mM) and HEPES (10 mM) buffers with increase in temperature from 20°C to 90°C.

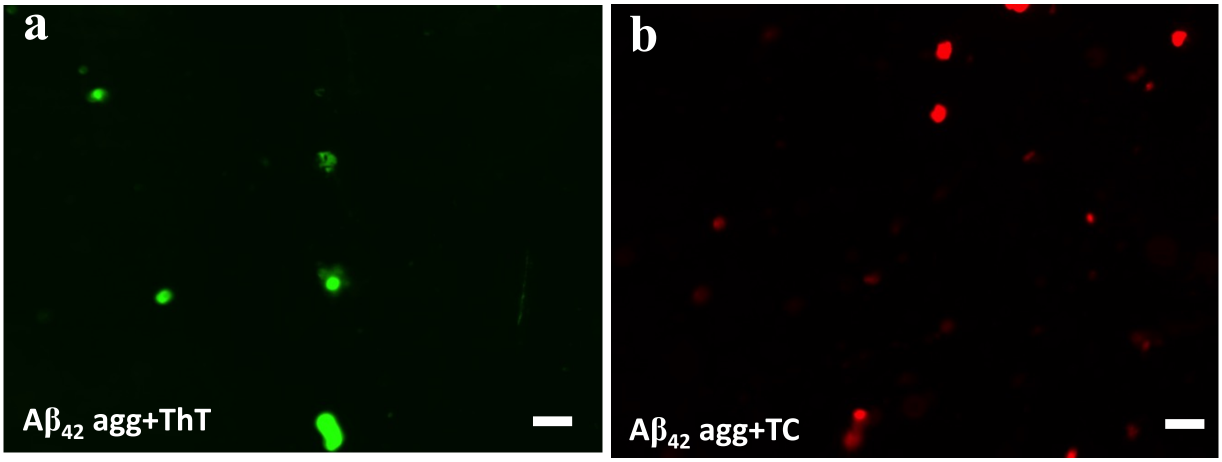

Figure 7. Fluorescence images of $A\beta_{42}$ fibrillar aggregates a) $A\beta_{42}$ fibrillar aggregates stained with ThT (control, show green fluorescence) and b) $A\beta_{42}$ fibrillar aggregates stained with **TC** show red fluorescence indicating switch on fluorescence capability of probe **TC** (scale bar 5 micron).

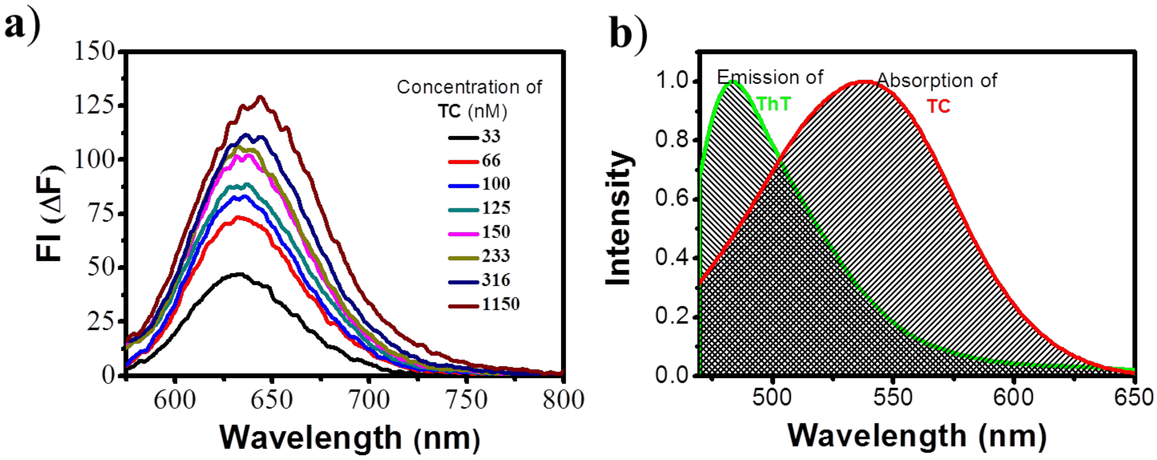

Figure S8. a) Difference fluorescence emission spectra of **TC** in PBS buffer and **TC** bound to $A\beta_{42}$ aggregates. Excitation wavelength $\lambda_{ex} = 537$ nm. Fluorescence intensity (FI) data was used for calculation of binding constant. b) Spectral overlap for emission from ThT and absorption of **TC**, showing favourable condition for FRET from ThT to **TC**.

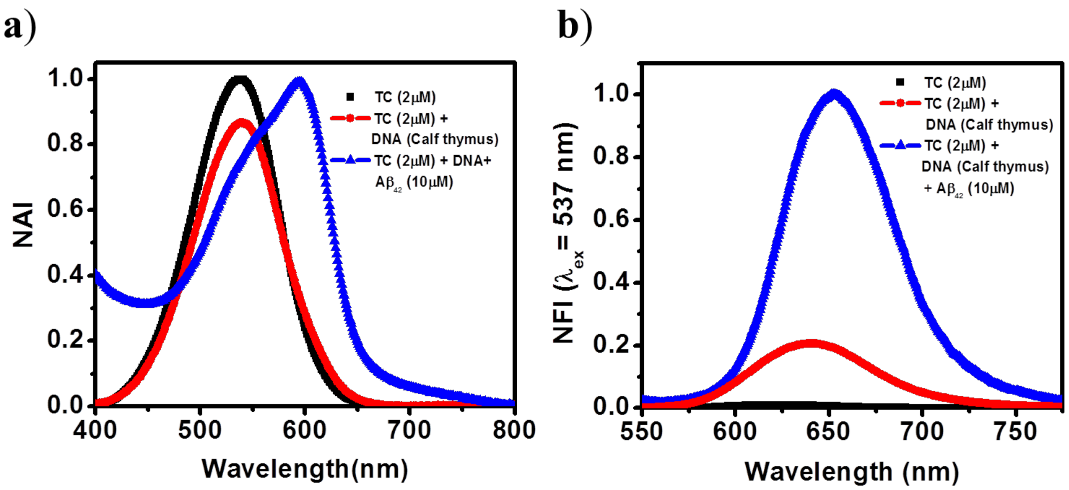

Figure S9. Competitive binding assay. a) Absorption spectra of probe **TC, TC** bound with DNA (Calf thymus) and addition of Aβ42 (10 μM) aggregates to the same sample, showing a red shift with a new band at 595 nm, normalized absorbance intensities (NAI). b) Emission spectra of probe **TC**, **TC** when treated with DNA (Calf thymus) and addition of Aβ42 (10 μM) aggregates to the same sample, shows better enhancement in fluorescence when compared to **TC** bound to DNA, normalized fluorescence intensities (NFI).

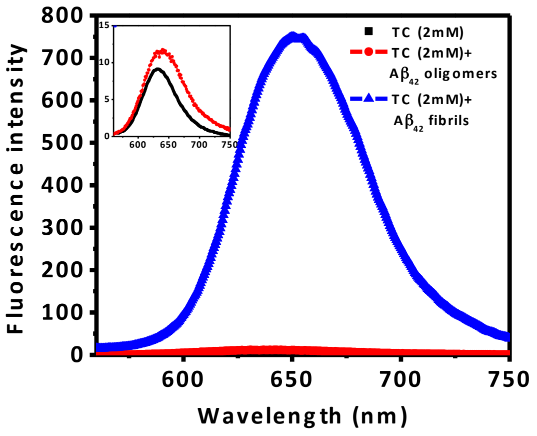

Figure S10. a) Emission spectra (λ_{ex}= 537 nm) of probe **TC** (2 μM), **TC** (2 μM) + Aβ42 oligomers (10 μM) and **TC** (2 μM) + Aβ42 fibrils (10 μM). Inset: shift in the emission maxima of probe **TC** upon interaction with Aβ42 oligomers and fibrils

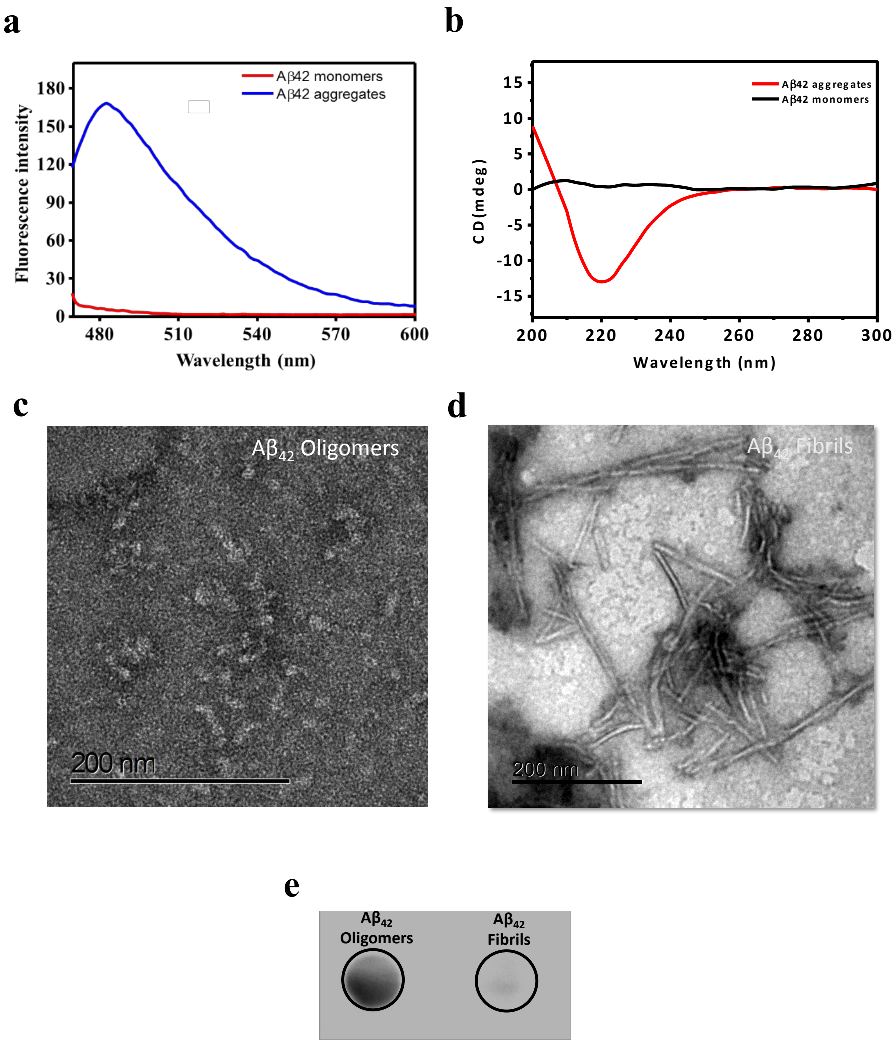

Figure S11. Thioflavin T, CD, TEM and dot blot analysis. a) Aβ42 aggregates analysed by thioflavin (ThT) assay. b) Aβ42 aggregates analysed by CD measurements c) TEM image of Aβ42 oligomeric species. d) TEM image of Aβ42 fibrillar aggregates. e) Dot blot was performed using A11 antibody (binds specifically to Aβ42 oligomers) for confirming the presence of Aβ42 oligomeric species.

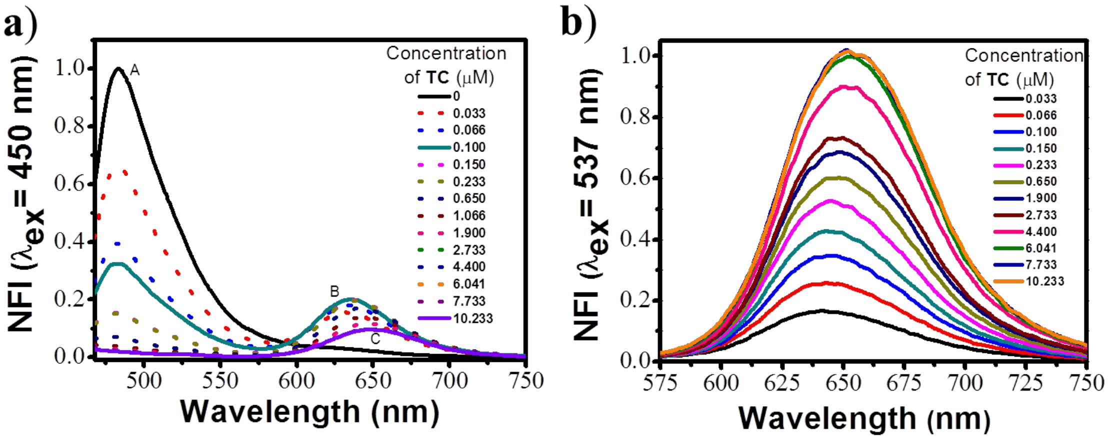

Figure S12. FRET and displacement assay. a) Normalized fluorescence intensities (NFI) of ThT and **TC** (λ_{ex} at 450 nm; fluorescence at 483 nm and ~654 nm) upon titration of a ThT/ Aβ_{42} aggregate complex (ThT, 5 μM/Aβ_{42} aggregates, 10 μM) with **TC**. b) Normalized fluorescence intensities (NFI) of **TC** (λ_{ex} at 537 nm; fluorescence measured at ~654 nm) upon titration of a ThT/ Aβ_{42} aggregate complex with **TC**.

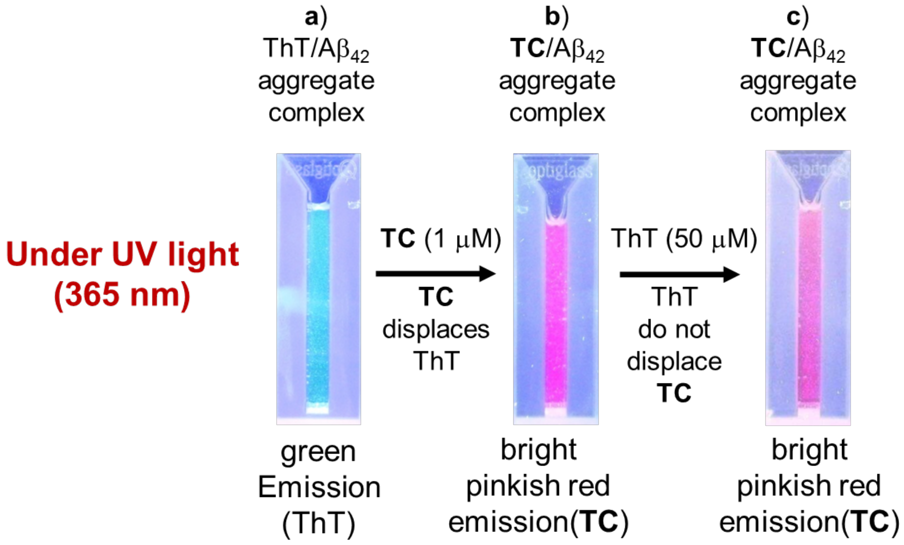

Figure S13. Photographs of ThT (10 μM)/ Aβ42 (50 μM) aggregates complex a), after adding **TC** (ThT displacement) b) and further addition of excess ThT (c) respectively, illuminated under UV light (365 nm).

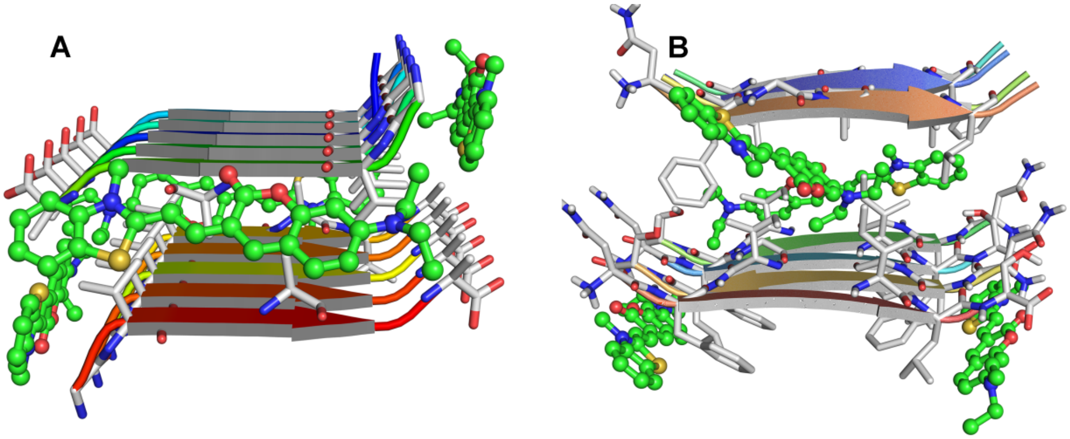

Figure S14. Docking mode of **TC** with α-synuclein (A) and IAPP (B) fibril. **TC** is shown in ball and stick mode, the protein in cartoon mode and the residues in contact with **TC** are in stick mode.

REFERENCES

(1) Nishimura, I., Hirano, A., Yamashita, T. & Fukami, T. Improvement of the high-speed logD assay using an injection marker for the water plug aspiration/injection method. *J. Chromatogr. A*, **1216**, 2984-2992 (2009).

(2) Lakowicz, J. R. *Principles of Fluorescence Spectroscopy 3rd ed.* Springer (2010).

(3) Murugan, N. A., Kongsted, J., Rinkevicius, Z. & Ågren, H. Break-down of the "first hyperpolarizability/ bond-length alternation parameter" relationship. *Proc. Natl. Acad. Sci. USA*, **107**, 16453-16458 (2010).

(4) Ågren, H. *et. al.* Hybrid density functional theory/molecular mechanics calculations of two-photon absorption of dimethylamino nitro stilbene in solution. *Phys. Chem. Chem. Phys.* **13**, 12506-12516 (2011).

(5) Murugan, N. A., Zalesny, R., Kongsted, J. & Ågren, H. Chelation-induced quenching of two-photon absorption of azacrown ether substituted distyryl benzene for metal ion sensing. *J. Chem Theory Comput.* **10**, 778-785 (2014).

(6) Murugan, N. A., Schrader, S. & Ågren, H. Role of protonation state and solvation on the pH dependent optical properties of bromocresol green. *J. Chem. Theor. Comp*. **10**, 3958–3968 (2014).

(7) Ågren, H. *et. al.* Association dynamics and linear and nonlinear optical properties of an N-acetylaladanamide probe in a POPC membrane. *J. Am. Chem. Soc,* **135**, 13590–13597 (2013).

(8) Frisch, M. J., Trucks, G. W., Schlegel, H. B., Scuseria, G. E., Robb, M. A., Cheeseman, J. R.; Scalmani, G., Barone, V., Mennucci, B., Petersson, G. A., Nakatsuji, H., Caricato, M., Li, X., Hrahian, H. P., Izmaylov, A. F., Bloino, J., Zheng, G., L. Sonnenberg, J., Hada, M., Ehara, M., Toyota, K., Fukuda, R., Hasegawa, J., Ishida, M., Nakajima, T., Honda, Y., Kitao, O., Nakai, H., Vreven, T., Montgomery, J. A., Peralta, J. E., Ogliaro, F., Bearpark, M., Heyd, J. J., Brothers, E., Kudin, K. N., Staroverov, V. N., Kobayashi, R., Normand, J., Raghavachari, K., Rendell, A., Burant, J. C., Iyengar, S. S., Tomasi, J., Cossi, M., Rega, N., Millam, J. M., Klene, M., Knox, J. E., Cross, J. B., Bakken, V., Adamo, C., Jaramillo, J., Gomperts, R., Stratmann, R. E., Yazyev, O., Austin, A. J., Cammi, R., Pomelli, C., Ochterski, J. W., Martin, R. L., Morokuma, K., Zakrzewski, V. G., Voth, G. A., Salvador, P., Dannenberg, J. J., Dapprich, S., Daniels, A. D., Farkas, Ö., Foresman, J. B., Ortiz, J. V., Cioslowski, J. & Fox, D. J., Gaussian, Inc., Wallingford CT, (2009).

(9) Ågren, H. *et. al. WIREs Comput. Mol. Sci*, **4**, 269–284 (2014).

(10) Peach, M. J. G., Benfield, P., Helgaker, T. & Tozer, D. J. Excitation energies in density functional theory: an evaluation and a diagnostic test. *J. Chem. Phys.* **128**, 044118 (2008).

(11) Handy, N. C. *et. al.* Assessment of a Coulomb-attenuated exchange–correlation energy functional. *Phys. Chem. Chem. Phys.* **8**, 558-562 (2006).

(12) Schrodinger, LLC (2010) The PyMOL Molecular Graphics System, Version 1.3r1
